# Transcriptome analysis reveals the protection mechanism of proanthocyanidins for *Saccharomyces cerevisiae* during wine fermentation

**DOI:** 10.1038/s41598-020-63631-2

**Published:** 2020-04-21

**Authors:** Jingyuan Li, Kaili Zhu, Hongwei Zhao

**Affiliations:** 1 College of Food Science and Engineering, Qingdao Agricultural University, Qingdao, Shandong Province 266109 China; 2 College of Marine Science and Bioengineering, Qingdao University of Science and Technology, Qingdao, Shandong Province 266042 China; 3 Shandong Provincial Key Laboratory of Biochemical Engineering, Qingdao, Shandong Province 266042 China

**Keywords:** Applied microbiology, RNA sequencing

## Abstract

Grape-derived proanthocyanidins could act as a protector against various environmental stresses for *Saccharomyces cerevisiae* during wine fermentation, resulting in the increased physiological activity, fermentation efficiency and improved wine quality. In order to explore the possible protection mechanism of proanthocyanidins globally, RNA-seq analysis for wine yeast AWRI R2 cultivated with 0 g/L (group A), 0.1 g/L (group B), 1.0 g/L (group C) proanthocyanidins were applied in this study. Differentially expressed genes were enriched into six metabolic pathways including vitamin B_6_, thiamine, amino acids, aminoacyl-tRNA, carbohydrate and steroid based on KEGG enrichment analysis. Four key genes (*SNZ2*, *THI6*, *THI21* and *THI80*), participated in the biosynthesis of vitamin B_6_ and thiamine, were up-regulated significantly in proanthocyanidins treated yeast cells and the gene expression levels were verified by RT-qPCR. Yeast cells supplemented with proanthocyanidins performed increased intracellular levels of vitamin B_6_ and thiamine and higher cell viability compared to the control group. In addition, the composition of intracellular fatty acids showed an obvious alternation in proanthocyanidins-treated yeast cells, in which the UFAs content increased whereas the SFA content decreased. In general, we provided an indirect protection effect of proanthocyanidins on the yeast cells to alleviate environmental stresses during wine fermentation.

## Introduction

The widely distributed phenolic substances in wine refer to a large group of chemical compounds that impact the sensory quality of red wine, such as color, flavor and astringency^1^. A large number of studies have shown that these phenolic substances have also biological activities of interest to the consumers, such as antioxidant properties, vascular protection and immune-modulatory effects^2–5^. For winemaking, these phenolic compounds are mainly extracted from grape skin, seed, and stem during the maceration and fermentation process^6^. Oenological practices, such as fermentation temperature, maceration time, storage period and yeast strains, are believed to impact the concentration and structure of these chemical compounds^7–9^.

As the predominant fraction, proanthocyanidins (PAs) account for more than 50% of the total wine polyphenols, and have several important roles in the quality of red wine, such as the perception of astringency and taste, aroma release, and color stability, throughout the entire period of fermentation and storage of wine^10,11^.

In addition, PAs could interact with other extracted phenolic compounds and even the fermentation strains, which have been proved to be crucial for the generation of new pigments, color and astringency modifications^12,13^. It has been reported that the interaction of the polysaccharides released by yeast lees with wine polyphenols during aging were crucial for sensory qualities of wines^11^. The extracted polyphenols could be adsorbed by yeast cells by forming the Van der Waals bonds and H-bonds during alcoholic fermentations, which might be a reasonable explanation that the yeasts cells could contribute to the modification of the color of the wine^13^.

*Saccharomyces cerevisiae*, the preferred microorganism for wine making and bioethanol production, has been exposed simultaneously and sequentially to a variety of environmental stresses during the fermentation process, including high concentration of ethanol, hyperosmotic stress, heat shock, low pH and nutrients deficiency^14–16^. Cells viability and fermentative behavior will be negatively affected by the stresses that in turn limit the wine production and quality^17^. In order to carry out wine fermentation properly, yeast cells should possess the capacity of rapid stress perceptions and responses to those stress conditions without substantial viability loss^14^. Therefore, to solve these problems, a number of previous studies have been performed in the aspects of robust strain isolation, genetic modification and fermentation process control^18–21^.

It has been reported that phenolic compounds in wine act as a structure-dependent and dose-dependent manner to affect the physiological activities and fermentation performance of yeast and bacterial cells^22–24^. Our previous work showed that PAs could pose obvious enhancement on the metabolism and fermentation efficiency, which has been elucidated from the metabolic level such as the change of glucose transport, the energy and redox homeostasis as well as the activities of rate-limiting enzymes in glycolysis^24,25^. However, the possible molecular mechanisms of the effects of PAs on the yeast fermentation performance have not been fully studied. Next-generation sequencing technologies enables the generation of genomic resources more efficient and cost-effective^26^. Therefore, in this study, we employed RNA-seq as the method to find the differentially expressed key genes and then performed comprehensive and comparative bioinformatics analysis to globally explore the possible protective effects of PAs on the yeast cells during wine fermentation.

## Results

### Quality evaluation of sequencing reads

Quality evaluation of sequencing reads needed to be evaluated before the following bioinformatics analysis. As shown in Table 1, a total of 524025062 clean data were generated with an average length of 147 bp. The total raw data was deposited in NCBI Gene Expression Omnibus (GEO) public archive database and the accession number is GSE141069. Phred quality score (Q score) was used as an index for the base calling accuracy and calculated by using the FastQC software (v0.10.1) based on Eq. (1):

1 $${\text{Q}}_{\text{phred}}=-\,10{\log }_{10}(\text{e})$$

**Table 1:** Quality statistics of clean sequencing data.

Sample	length	Reads	Bases	Q20 (%)	Q30 (%)
A1	147.32	40811558	6012389770	95.73	88.78
A2	147.38	62030546	9141997823	95.75	89.47
A3	147.53	60816138	8972430801	95.93	89.89
B1	147.45	67018594	9882123005	95.88	89.72
B2	147.49	55217110	8143748191	96.01	90.04
B3	147.41	66504646	9803127605	95.89	89.78
C1	147.18	52310564	7699225784	95.85	89.70
C2	147.28	55806604	8219088002	95.85	89.69
C3	147.21	63509302	9349372301	96.02	90.10

Q20, Q30 refer to the proportion of base calls with Phred scores >20 or 30 in the total bases. Higher scores ensured the base calling accuracy and data quality. Each group has three biological replicates.

About 90% of the clean reads in each sample has Phred quality score >30, indicating that the base calling accuracy for each group was 99.9% and met the requirement for the next analysis (Table 1).

The Pearson correlation coefficients R^2^ were calculated to assess the reproducibility of gene expression values across biological replicates, as shown in Supplementary Fig. S1 to Supplementary Fig. S3, the average correlation R^2^ across the replicates for each group (A, B, C) were 0.969, 0.967 and 0.931, respectively, indicating that there were no considerable differences between the biological replicates and the data could be used in following analysis.

### Differential gene expression analysis

**Figure 1:**
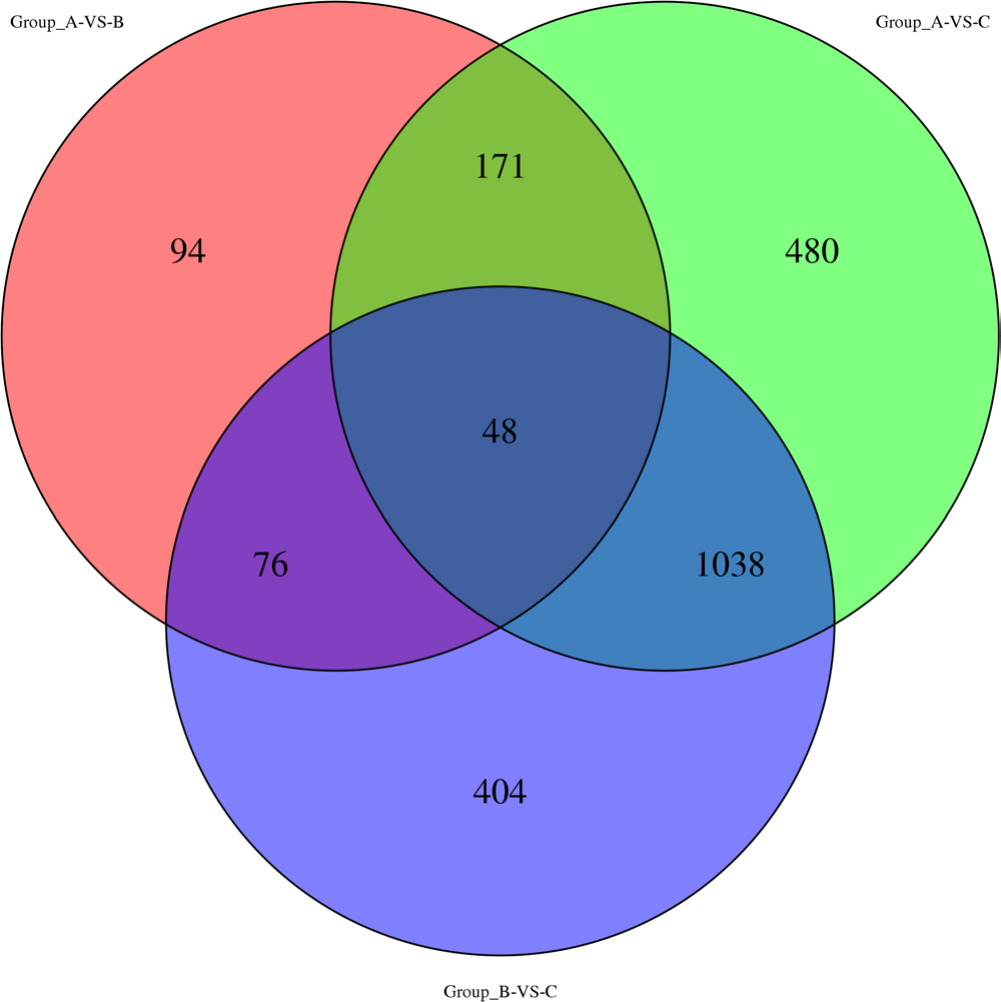
Venn diagrams of transcripts in the three comparative groups marked as group A versus group B, group A versus group C and group B versus group C, respectively.

**Figure 2:**
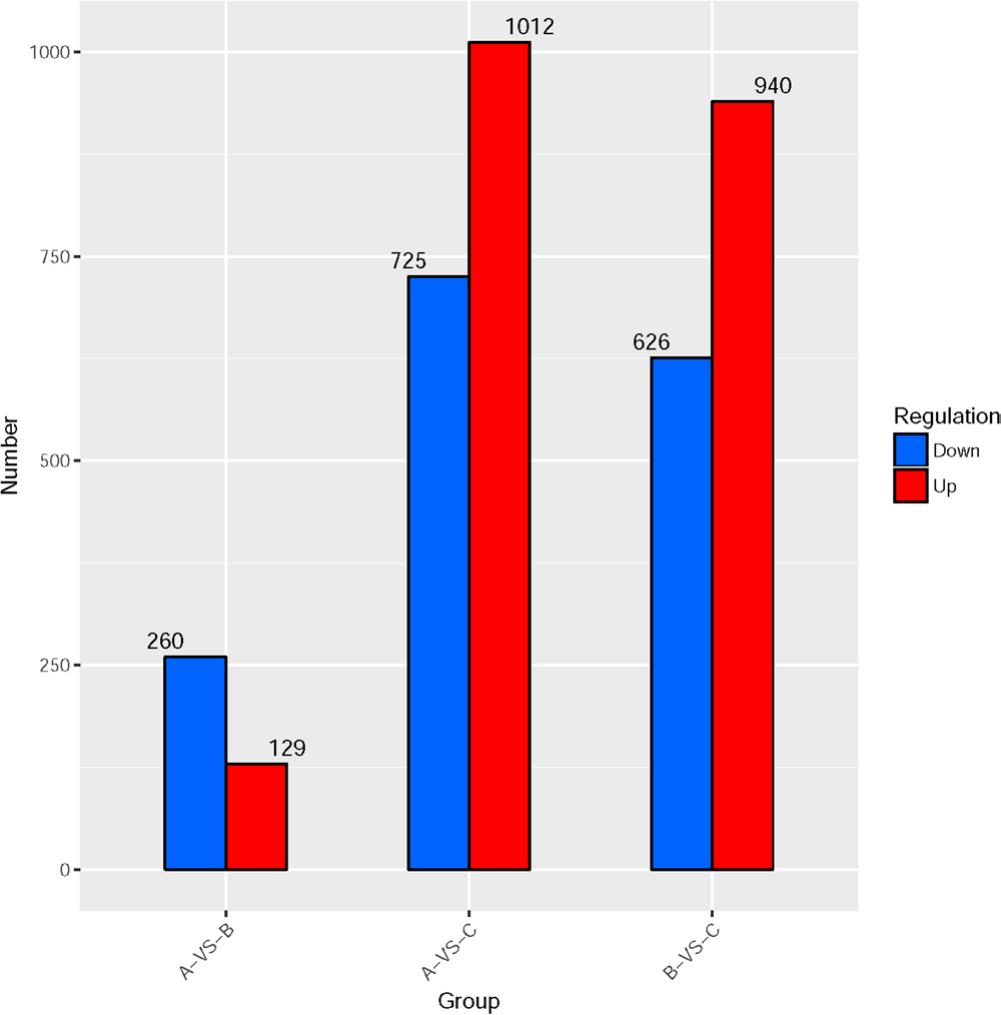
Up or down regulated DEGs in pairwise comparisons marked as group A versus group B, group A versus group C and group B versus group C, respectively.

To identify differentially expressed genes, transcriptome analysis was carried out for the yeast cells supplemented with 0 g/L (group A), 0.1 g/L (group B) and 1.0 g/L (group C) PAs, respectively. As shown in Fig. 1, 389, 1737 and 1566 DEGs were identified by comparative analysis of transcriptome data of group A versus group B, group A versus group C and group B versus group C, respectively, indicating that PAs pose considerable effects on the gene expression level in the yeast cells. Moreover, a dose-dependent increase of DEGs was found in the data comparison (Fig. 2). With the increase of the concentration of PAs from 0.1 g/L to 1.0 g/L in the fermentation broth, more than 4 times of DEGs were identified in the yeast cells. A total of 48 DEGs were found in all the three comparative groups, which might be considered as the key genes related to the PAs protection mechanism.

### GO enrichment analysis of the DEGs

**Figure 3:**
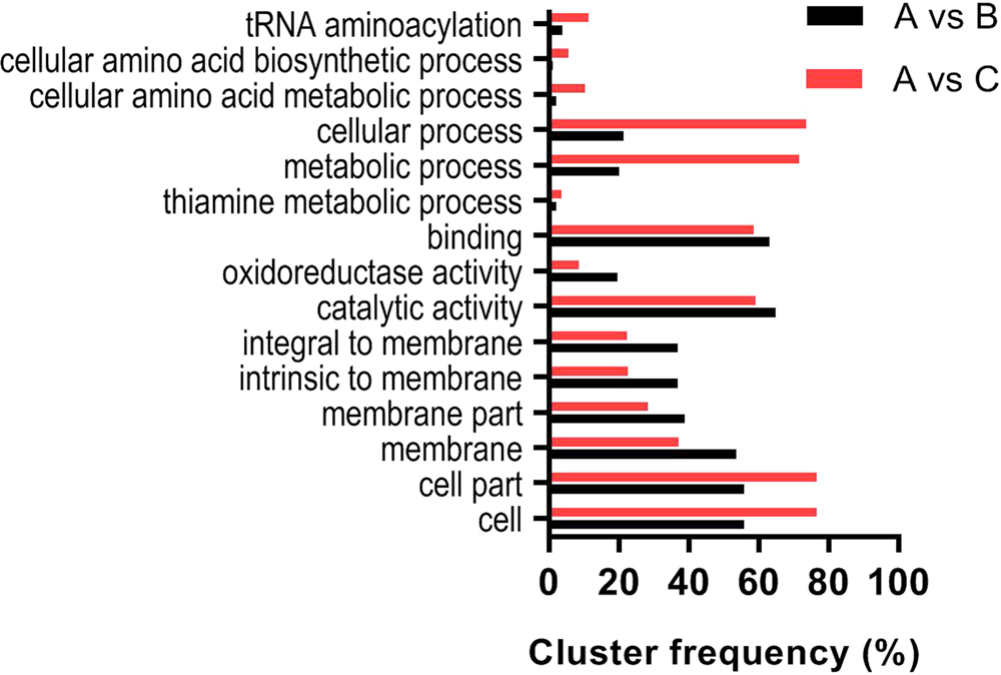
Gene ontology (GO) analysis of the identified DEGs.

To better understand the functions of the DEGs, gene annotation including homologous protein annotation was performed by using the gene ontology (GO) database. As shown in Fig. 3, the identified DEGs could be classified into three categories including biological process, cellular component and molecular function.

In the cellular component category, DEGs were mainly enriched in the terms related with the structure of cell membrane and organelle; In the biological process group, metabolic process and cellular process were the important functions, especially the thiamine and cellular amino acid metabolic pathways and tRNA aminoacylation process; catalytic activity, oxidoreductase activity and binding were the three main molecular functions of the identified DEGs.

In addition, an obvious positive correlation between the number of enriched DGEs and the concentration of PAs were found in the three biological functions. Especially in the thiamine metabolic process and tRNA aminoacylation, as the concentration of PAs was increased to 1.0 g/L, more than 6 times of the numbers of DGEs were enriched compared to that in the 0.1 g/L PAs-treated group. It indicated that PAs could act as an exogenous regulator to perturb more genes expression to protect yeast cells against the stress factors during fermentation.

### Metabolic pathways enrichment analysis

To explore the basic protection mechanism of PAs in yeast cells, the main metabolic or signaling pathways were enriched using the KEGG database (Table 2). In the comparison between PAs treated and untreated groups, six pathways, including vitamin B_6_ metabolism, thiamine metabolism, biosynthesis of amino acids, aminoacyl-tRNA biosynthesis, starch and sucrose metabolism and steroid biosynthesis were significantly enriched (p values were shown in Table 2). Most of the enriched genes within the identified pathways were up-regulated significantly (p values were shown in Table 2), indicating that PAs could be act as an extracellular enhancer to regulate the important metabolic pathways to produce more anabolic products (e.g. vitamins, amino acid, glucan, ergosterol and fatty acid) necessary to survive in wine fermentation process.

**Table 2:** The KEGG pathway enrichment analysis.

Gene name	Description	log2 Fold Change	Corrected p-value
**Vitamin B**_6_ **metabolism**			**4.54×10**^**−4**^
*SNO3*	putative pyridoxal 5′-phosphate synthase	+1.01	1.08×10^−5^
*SNZ2*	GMP synthase activity	+1.46	3.15×10^−40^
*SNZ3*	Pyridoxal-5′-phosphate synthase	+1.48	1.34×10^−28^
*SNO2*	putative pyridoxal 5′-phosphate synthase	+1.75	2.67×10^−40^
**Thiamine metabolism**			**8.15×10**
*THI80*	thiamine diphosphokinase activity	+1.23	4.34×10^−20^
*THI21*	phosphomethylpyrimidine kinase activity	+1.54	7.4×10^−18^
*THI5*	Protein involved in synthesis of the thiamine precursor HMP	+1.08	1.39×10^−4^
*THI6*	thiamine-phosphate diphosphorylase activity	+1.21	9.36×10^−18^
*THI20*	phosphomethylpyrimidine kinase activity	+1.14	2.12×10^−13^
*THI22*	hydroxymethylpyrimidine phosphate kinases	+1.39	1.51×10^−15^
*THI72*	Transporter of thiamine or related compound	+1.12	5.67×10^−10^
**Arginine and proline metabolism**			**6.43×10**
*CAR1*	arginase	−2.10	2.28×10^−32^
*CAR2*	L-ornithine transaminase	+1.04	4.91×10^−8^
*PUT2*	δ−1-pyrroline-5-carboxylate dehydrogenase	−1.00	1.44×10^−10^
*PUT4*	Proline permease	−5.40	1.72×10^−147^
*PRO2*	γ-glutamyl phosphate reductase	−1.26	3.12×10^−17^
*GAP1*	Amino acid permease	−2.09	3.23×10^−18^
*SPE1*	Ornithine decarboxylase	−1.23	3.73×10^−15^
**Aminoacyl-tRNA biosynthesis**			**5.09×10**
*MSE1*	glutamate-tRNA ligase activity	+1.84	5.04×10^−50^
*MSW1*	tryptophan-tRNA ligase activity	+1.38	1.34×10^−32^
*MSM1*	methionine-tRNA ligase activity	+1.69	6.71×10^−32^
*MSF1*	phenylalanine-tRNA ligase activity	+1.60	1.76×10^−30^
*MSD1*	aminoacyl-tRNA ligase activity	+1.34	9.97×10^−28^
*AIM10*	proline-tRNA ligase activity	+1.42	3.10×10^−25^
*HER2*	glutaminyl-tRNA synthase activity	+1.12	3.00×10^−17^
*DIA4*	serine-tRNA ligase activity	+1.56	8.10×10^−17^
*TYS1*	tyrosine-tRNA ligase activity	−1.42	8.10×10^−17^
*KRS1*	lysine-tRNA ligase activity	−1.34	5.02×10^−9^
*CDC60*	leucine-tRNA ligase activity	−1.42	5.11×10^−9^
*THS1*	threonine-tRNA ligase activity	−1.20	6.05×10^−9^
*DPS1*	aspartate-tRNA ligase activity	−1.15	1.05×10^−8^
*DED81*	asparagine-tRNA ligase activity	−1.20	1.17×10^−8^
*ALA1*	alanine-tRNA ligase activity	−1.19	6.36×10^−8^
*FRS1*	phenylalanine-tRNA ligase activity	−1.01	4.96×10^−7^
*GLN4*	glutamine-tRNA ligase activity	−1.03	9.35×10^−5^
**Starch and sucrose metabolism**			**4.56×10**
*GSC2*	1,3-beta-D-glucan synthase activity	+1.89	1.43×10^−9^
*DSE4*	glucan endo-1,4-beta-glucanase activity	+1.36	8.55×10^−21^
**Steroid biosynthesis**			**1.48×10**
*ERG26*	C-3 sterol dehydrogenase	+1.14	1.55×10^−11^
*ERG5*	oxidoreductase activity	+1.26	7.52×10^−8^
*ERG3*	oxidoreductase activity	+1.25	2.11×10^−7^
*ERG4*	oxidoreductase activity	+2.13	6.11×10^−23^
*ERG28*	ergosterol biosynthesis	+1.77	2.81×10^−10^

The column of “log2 Fold Change” represents the base-two logarithm value of the fold change of the gene expression; “+” means up-regulated genes; “−” means down-regulated genes. Hypergeometric test was used for statistical analysis. The p-values have been corrected for multiple testing by the Benjamini and Hochberg adjustment method. The corrected p value <0.05 plus the absolute log2 Fold Change value >1 were considered statistically significant.

### Quantitative Real-time PCR

**Figure 4:**
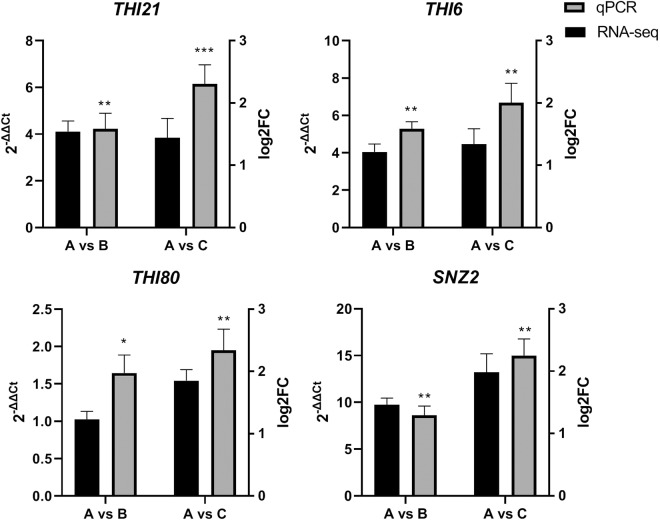
RT-qPCR validations of the results obtained in RNA-seq analysis. The relative expression level of 4 selected genes compared to the actin gene *ACT1* was calculated. Value of the 2^−ΔΔCT^ was calculated and two-way ANOVA with Tukey’s multiple comparisons test were used for the significant analysis of the RT-qPCR at *p  <  0.05, **p  <  0.01, ***p  <  0.001 level. The absolute log2 Fold Change value >1 were considered statistically significant in the RNA-seq analysis. Three biological replicates were prepared and the data were shown as means ± S.D.

To further confirm the alterations of gene expressions in PAs-treated yeast cells, we measured the relative expression level of four DEGs related to the metabolic pathway of B-complex vitamins (B_1_, B_6_) by RT-qPCR (Fig. 4). The results of RT-qPCR showed a good consistence with that obtained in RNA-seq analysis. The expression levels of the selected genes were up-regulated significantly in PAs-treated groups as relative fold change to the control. More interestingly, a dose-dependent up-regulation appeared as the concentration of PAs increased to 1.0 g/L. Therefore, the RNA-seq could provide reliable data for differential gene expression analysis.

**Figure 5:**
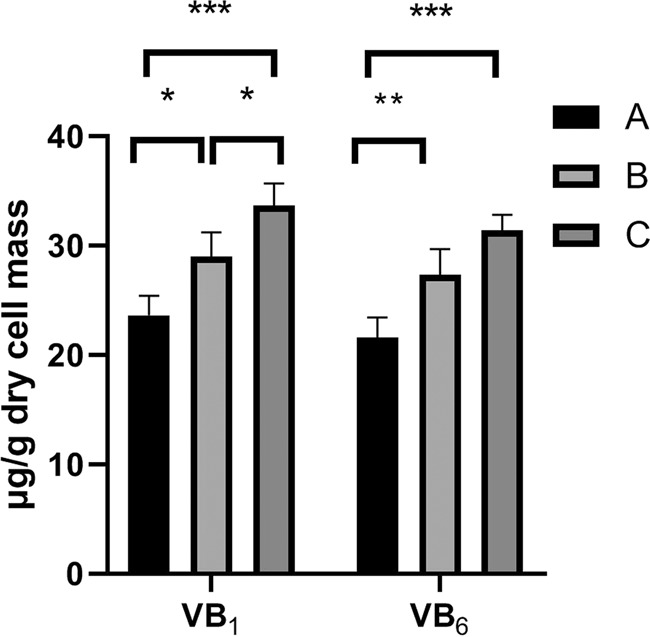
Intracellular contents of vitamins B_1_ and vitamins B_6_ in different systems. All data represent the mean values ± S.D. from three independent experiments. Two-way ANOVA with Tukey’s multiple comparisons test were used for the significant analysis at *p  <  0.05, **p  <  0.01, ***p  <  0.001 level.

Based on the results of transcriptome analysis, the genes related to the metabolic pathway of B-complex vitamins (B_1_, B_6_) were up-regulated significantly. In addition, the contents of vitamins B_1_ and vitamins B_6_ were measured to verify the impact of PAs from the aspect of yeast cells phenotype (Fig. 5). Calibration curves of peak area-concentration relationship for each vitamin were prepared using a series of mixed solutions at range from 1.0 to 5.0 μg/ mL. As shown in Supplementary Fig. S4, it showed a good linear relation with correlation coefficients (R^2^) obtained as 0.9947 and 0.9987 for vitamins B_1_ and B_6_ at the selected concentration ranges, respectively. This method was suitable for the calculation of the intracellular contents of the vitamins B_1_ and B_6_. As shown in Fig. 5, significantly increases of the contents of vitamins B_1_ and vitamins B_6_ were detected in PAs-treated yeast cells. For example, vitamins B_1_ and vitamins B_6_ contents in yeast cells treated with 0.1 g/L PAs increased significantly from 23.6 μg/g dry weight and 21.6 μg/g dry weight to 28.9 μg/g dry weight (p = 0.015) and 27.4 μg/g dry weight (p = 0.001), respectively. In addition, like the elevation in gene expression levels, obvious dose-dependent increases of intracellular level of vitamins B_1_ and vitamins B_6_ in the PAs-treated yeast cells were observed, especially for the vitamins B_1_ contents which showed a 16.6% increase in the 1.0 g/L PAs-treated yeast cells compared to the 0.1 g/L PAs-treated ones.

**Figure 6:**
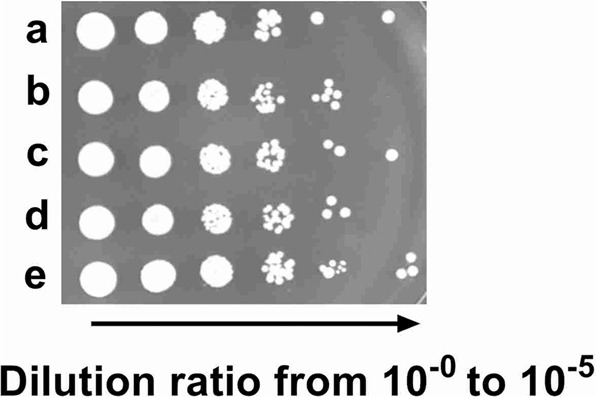
Spot assay of the viability of yeast cells treated with 1.0 g/L PAs (b), 50 mg/L vitamin B_1_ (c), 50 mg/L vitamin B_6_ (d) and a 1:1(w/w) mixture of the two vitamins (e), respectively in MSM medium. The negative control (a) was set without the above supplements. After cultivation at 28 °C for 72 h, five microliter of each proper dilution was spotted on the MSM agar plate for viable cell counts. The arrow below the panel indicates a serial dilution of plated cells (1:1; 1:10; 1:10^2^; 1:10^3^; 1:10^4;^ 1:10^5^ from left to right).

To further prove the protection effects of PAs on the yeast cells, measurements of the cell viability were conducted. The yeast cells were cultivated with 1.0 g/L PAs, 50 mg/L vitamin B_1_, 50 mg/L vitamin B_6_ and a 1:1(w/w) mixture of the two vitamins, respectively in MSM medium at 28 °C for 72 h, and then the viable cells were counted on the MSM agar plates. As shown in Fig. 6, cell survival decreased significantly in the negative control group in which no PAs or vitamins were supplemented. As expected, the viable cells on plates containing PAs, vitamin B_1_, vitamin B_6_ and mixture of vitamin B_1_ and B_6_, respectively, were higher than the control group. Combined with the results in Figs. 4 and 5, we could clearly notice that exogenous PAs could increase the bio-synthetic yields of vitamin B_1_ and vitamin B_6_ to relieve the environmental stresses in yeast cells during red wine fermentation.

**Figure 7:**
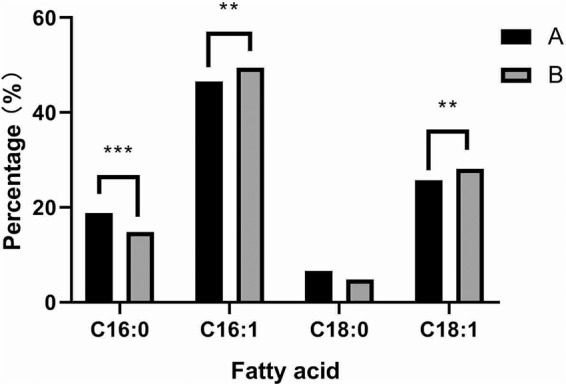
Fatty acid profiles in the membrane of yeast cells. *S. cerevisiae* yeast strains AWRI R2 were cultivated statically at 28 °C for 72 h with (grey bars) or without (dark bars) the treatment of PAs. And then membrane fatty acid compositions were measured. Data were shown as the mean ± S.D. of three independent experiments. Two-way ANOVA with Tukey’s multiple comparisons test were used for the significant analysis at *p  <  0.05, **p  <  0.01, ***p  <  0.001 level.

It has been reported that yeast membrane fatty acid composition and membrane fluidity were linked to the stress tolerance of the cells. Exogenous ergosterol has been proved to be effective for the enhancement of the tolerance of monoterpenes in *S. cerevisiae* strain by regulating the fatty acid composition^27^. The intracellular contents of vitamin B_1_ and vitamin B_6_ could pose significant effects on the lipid metabolism. To testify whether the PAs show the similar effects on alternation of lipid metabolism and yeast tolerance for environmental stresses, we studied the changes in fatty acid profile in the yeast cells with or without the treatment of PAs. As shown in Fig. 7, lipid profile of PAs-treated cells revealed a 5.36% increase in cellular unsaturated fatty acids (C16:1 and C18:1) with respect to cells grown in the absence of PAs. This comprised a 2.93% increase in C16:1 and a 2.43% increase in C18:1. Meanwhile, the saturated fatty acids (C16:0 and C18:0) in the PAs-treated cells decreased significantly from 25.4% to 19.6% of the total phospholipid fatty acid compared to that in non PAs-treated cells.

## Discussion

Generally speaking, red wine fermentation process could be considered as the interaction of yeast cells and the complex composition of the grape, especially the sugar, organic acid and phenolic compounds^28^. Sensory attributes of wine including color intensity and stability, mouth-feel, astringency and taste mainly come from the phenolic compounds evolution, especially PAs, the principal constitute in the phenolic compounds^29^. In order to yield high quality red wine with improved sensory and biological properties, thoroughly studies of interactions between yeast cells and PAs should be done. In our previous studies, we have showed that the concentration and structure of PAs evolved progressively in a strain- and time-dependent manner during fermentation^1^. Moreover, PAs could pose positive effects on the yeast cells that have proved from the aspects of glucose transport, the energy and redox homeostasis as well as the activities of rate-limiting enzymes in glycolysis^24^. However, to the best of our knowledge, the global regulation posed by PAs from the genetic and metabolic level has never been reported before.

With the rapid development of sequencing technology, RNA-seq has become more efficient and cost-effective in recent years^26^. Identifying key genes and metabolic pathways related to the stress-tolerance abilities of *S. cerevisiae* has been considered as the basis of understanding the intracellular global regulation patterns and making the wine fermentation process more controllable^30^. Therefore, in the present study, we employed the RNA-seq as well as bioinformatics analysis to uncover the possible protection mechanism of PAs for *S. cerevisiae* during wine fermentation.

We identified a great many of DEGs by measuring the expression levels in the yeast cells cultivated with 0 g/L (group A), 0.1 g/L (group B), 1.0 g/L (group C) PAs, respectively. By GO and KEGG enrichment analysis, the gene functions and participated metabolic pathways have been uncovered. The significantly enriched gene functions were about the structure of cell membrane, thiamine metabolic processes, cellular amino acid metabolic processes, tRNA amino acylation process, oxidoreductase activity and binding activity. The identified DEGs were significantly enriched in to six metabolic pathways including vitamin B_6_, thiamine, amino acids, aminoacyl-tRNA, carbohydrate and steroid. Most of the enriched genes in the metabolic pathways of vitamin B_6_, thiamine, starch and sucrose and steroid showed significantly up-regulated expression patterns compared to that of control group, indicating PAs could act as an extracellular activator to enhance the metabolic efficiency of vitamin B_6_, thiamine, carbohydrates and steroids.

In our study, for the first time we showed that the genes (*SNO3*, *SNZ2*, *SNZ3*, *SNO2*, *THI80*, *THI21*, *THI6*, *THI20, THI72, THI5, THI22*) in the PAs-treated yeast cells performed significantly higher expression levels, involving in the sequentially enhanced bio-synthesis of vitamin B_1_ and B_6_ for yeasts (Figs. 4 and 5). It has been reported that increased amplification number of these genes related to bio-synthesis of vitamin B_1_ and vitamin B_6_ confers the efficient growth of yeast cells in the thiamin and/or pyridoxine-deficient medium with high sugar concentrations^31^. Interestingly, our results consistent with the previous study that the yeast cells supplemented with PAs and vitamin B_1_ and B_6_ compounds showed higher viability compared to the control group (Fig. 6). It deduced there must be problems with the bio-availability of these vitamins in the wine making process and exogenous PAs could cover the deficiency of the two important vitamins for yeast cells against environmental stresses^32^. It also has reported that decreased intracellular vitamin B_6_ content could repress many metabolic pathways, including reduced respiration activity and a significant alteration in sterol and unsaturated fatty acids metabolism^33^. It might resulted in the sequential changes in the composition of lipids, affecting the fluidity and permeability of cell membrane, as wells as the cell growth^27^. In our study, we also studied the fatty acid profiles in the membrane of yeast cells with or without the treatment of PAs. A significant increase in the quantities of unsaturated fatty acids (C16:1 and C18:1) was found in the presence of PAs compared to the control group, whereas the quantities of palmitic acid (C16:0) and stearic acid (C18:0) decreased significantly, reflecting that an indirect interaction between PAs and the metabolisms of fatty acid and vitamin B_6_ might exist in yeast cells by which the phenolic compounds in wine could enhance the physiological activities and fermentation performance. Higher contents of unsaturated fatty acids in yeast cells membranes could make the yeast cells less sensitive to heat stress by repressing the expression level of genes involving the heat-shock response (HSR) and general stress response (GSR) pathways^34^. In other words, there was an obvious positive correlation between the contents of unsaturated fatty acids and stress tolerance capacity of yeast strains. Our findings provided an exciting possibility that it might be possible to make yeast cells more robust by simply modifying the percentage of PAs in the medium.

Thiamine is another essential molecule for all living organisms. Deficiency in thiamine could decrease NADPH, which affected cellular anabolism and lead to a cessation of glucose consumption^35^. As a cofactor of transketolase in pentose phosphate pathway, thiamine also could accelerate the metabolism from fructose 6-phosphate and glyceraldehyde 3-phosphate to ribulose-5-phosphate and erythritol 4-phosphate, precursors for the synthesis of amino acids in yeast cells under stress situations^36^. Our previous study showed that PAs could pose significant enhancement on the activity of H^+^-ATPase, cell growth, and alcoholic fermentation rate by up-regulating the gene expression levels of *PMA1* and *HXT7*, encoding the plasma membrane ATPase and yeast hexose transporter, respectively^24,25^. That is, the benefits provided by exogenous PAs to the growth and fermentation performances of yeast cells could be explained from the interaction between the phenolic substances in wine and the metabolisms of thiamine and amino acid.

In our study, the bio-synthesis pathway of ergosterol was also significantly enriched that the expression of five related genes (eg. *ERG3*, *ERG4*, *ERG5*, *ERG26*, *ERG28*) were highly induced in the yeast cells with the treatment of PAs. Ergosterols are essential structural and regulatory components involving in the fluidity and permeability of yeast cells membrane. Higher contents of ergosterol could protect yeast against multiple environmental stresses such as oxidative, ethanol, osmotic and so on^37^. Enhanced amplification numbers of ergosterol synthesis-related genes in yeast cells have been proved to be connected with the PAs supplementation (Table 2). Exogenous PAs might affect the contents of ergosterol and in sequentially contribute for the enhancement of stress tolerance ability in the yeast cells which should be further verified in the future.

It has been reported accumulation of amino acids in plant or microorganisms was critical for the environmental stresses tolerance. The accumulated amino acids could act as osmoprotectants, ROS scavenger and protector against heavy metals^38–40^. The possibility is that free amino acid could interfere with the side chain bonding and introduce conformational changes in the enzyme protein and thus affect the enzymes synthesis and activity^41^. Our data demonstrated the pathways of arginine and proline metabolism and aminoacyl-tRNA biosynthesis were significantly enriched in PAs-supplemented yeast cells by the KEGG analysis. Arginine and proline were considered as the important osmoprotectant, playing pivotal role of metabolism in cellular osmoregulation^42^. The expression level of genes involving in the arginine and proline metabolism showed up or down-regulated patterns (Table 2). *CAR2* gene expression level was up-regulated significantly by feeding with PAs, and it might contribute to more synthesis of L-ornithine transaminase which could catalyze glutamate-γ-semialdehyde (GSA) to ornithine in cytosol, the precursor of arginine, more efficiently and alleviate the encountered environmental stress^43^. The uptake for proline in yeast was largely mediated by a high affinity, specific permease, Put4p, and a low affinity general amino acid permease, Gap1p, encoded by *PUT4* and *GAP1*, respectively^44^. However, in our study, the gene expression level of *PUT4* and *GAP1* in the PAs-treated yeast cells showed significant down-regulated. The two inconsistent metabolic patterns of arginine and proline might due to the species-specific alternation or the PAs regulation which should be further studied. Other down-regulated genes (*CAR1*, *SPE1*, and *PUT2*) repressed the degradation pathways of the two kinds of protective amino acids. The gene *PRO2* (encoding γ-glutamyl phosphate reductase) were involving in the catalysis of the second step in proline biosynthesis from glutamate. Glutamate and proline were both important amino acid in protecting yeast cells against stress factors. Pyrroline-5-carboxylate (P5C) was an critical reaction intermediate making it possible of the interconversion of glutamate, ornithine, proline and arginine^45^. In PAs-treated yeast cells the gene expression level of *PRO2* was down-regulated significantly, reflecting the cells decreased the consumption of glutamate for the accumulation of proline. It might deduce the phenolic compounds could act as a regulator for the dynamic balance of the intracellular amino acids in yeast cells during wine fermentation.

During wine fermentation, a global regulation is required to maintain the intracellular homeostasis for the survival and growth of the yeast cells. All of the physiological activities could be considered as the enzymatic reactions. Therefore, it was vital that translation reprogramming should be in a correct and controllable way^46^. In our study, a large amount of genes involving in the aminoacyl-tRNA biosynthesis were differential expressed significantly in PAs-treated yeast cells during fermentation. It indicated that the phenolic compounds in wine could regulate the biosynthesis and activity of aminoacyl-tRNA ligase, and subsequently affect the protein translation globally as the cells responded to the environmental stresses to maintain the intracellular homeostasis.

## Conclusions

RNA-seq analysis was employed in our study to systematically explore the protection mechanism of PAs on the yeast cells during wine fermentation. A large amount of DEGs were identified and subsequently enriched into six metabolic pathways including vitamin B_6_, thiamine, amino acids, aminoacyl-tRNA, carbohydrate and steroid. The intracellular contents of the thiamine and vitamin B_6_ increased significantly in PAs-treated yeast cells, which was in consistent with the cell viability and the expression pattern of the identified DEGs participating in the metabolic pathways of vitamin B_6_ and thiamine. In addition, it revealed a significant difference in the fatty acid composition between the PAs-treated yeast cells and the control that the UFA (C16:1 and C18:1) increased whereas SFA (C16:0 and C18:0) decreased. The other enriched metabolic pathways in RNA-seq analysis such as the biosynthesis of amino acids, proteins and steroids should be mainly focused on in further study to fully elucidate the interactions between the phenolic compounds in wine and the yeast cells. In general, data of this study provided insights into the possible molecular mechanisms of PAs protection in *S. cerevisiae* yeast strains during wine fermentation process and might contribute to the selection and modification of yeast strains with enhanced resistance capacity.

## Materials and methods

### Yeast strain and culture conditions

AWRI R2, used in this study, was a commercial yeast strain from Marivin (Queensland, Australian) and has good fermentation performance. Yeast cells were maintained on YPD medium slants (1% yeast extract, 2% peptone, 2% glucose, 2% agar). Fresh yeast inoculum was prepared by transferring one loop of yeast colony into 100 mL YPD medium in a 250 mL flask and incubating aerobically overnight in a shaker at 28 °C and 120 rpm.

### Fermentation

Model synthetic medium (MSM) was used for fermentation in this study. The MSM is a chemically well-defined medium to simulate a standard grape juice for wine fermentation in laboratory^47^. The composition of this medium was as follows: glucose (100 g), fructose (100 g), tartaric acid (3 g), citric acid (0.3 g), L-malic acid (0.3 g), MgSO_4_ (0.2 g), KH_2_PO_4_ (2 g), (NH4)_2_SO_4_ (0.3 g), asparagine (0.6 g), MnSO_4_·H_2_O (4 mg), ZnSO_4_·7H_2_O (4 mg), CuSO_4_·5H_2_O (1 mg), KI (1 mg), CoCl_2_·6H_2_O (0.4 mg), (NH_4_)_6_Mo_7_O_24_·4H_2_O (1 mg), H_3_BO_3_ (1 mg), meso-inositol (300 mg), biotin (0.04 mg), thiamin (1 mg), pyridoxine (1 mg), nicotinic acid (1 mg), pantothenic acid (1 mg), p-amino benzoic acid (1 mg), palmitic acid (1 mg), palmitoleic acid (0.2 mg), stearic acid (3 mg), oleic acid (0.5 mg), linoleic acid (0.5 mg) and linolenic acid (0.2 mg). The MSM was prepared by mixing thoroughly the above components in 1 L deionized water and the final pH value of the medium was adjusted to 3.3. The crude PAs was firstly subjected to a column-based purification procedure according to our previous research protocol^25^ to remove the monomers and oligomers. The purified PAs was weighted accurately and dissolved in 10 mL absolute ethyl alcohol thoroughly before use. The obtained PAs solutions were added into the MSM medium to make a series of final concentrations of 0.1 g/L, 1.0 g/L (marked as group B and group C, respectively). The control group marked as group A was prepared by adding 10 mL absolute ethyl alcohol without PAs into the fermentation medium directly. The prepared culture media were sterilized by filtration (nitrate cellulose membrane, 0.45 μm) and supplemented with sulfur dioxide (20 mg/L) to simulate the oenological environment before yeast strain inoculation.

For the fermentation, one percent of the prepared yeast inoculum was inoculated into 400 mL MSM medium supplemented with 0, 0.1 and 1.0 g/L PAs in 500 mL flasks equipped with glass capillary stoppers to maintain the anaerobic conditions and cultivated statically at 28 °C. Samples were taken at the 72 h that the yeast cells were staying in the stationary phase. Ten milliliter of the fermentation broth was centrifuged at 6000 r/min for 5 min to collect the yeast cell pellets. The obtained yeast cells were washed 3 times with 0.9% sodium chloride solution and rapidly frozen in liquid nitrogen before stored at −80 °C for the following experiments.

### RNA extraction and sequencing

Yeast cells were grinded in liquid nitrogen. Total RNA of each sample was extracted using RNeasy Mini Kit (Qiagen, Germany). The obtained RNA products were quantified and qualified by Agilent 2100 Bioanalyzer (Agilent Technologies, Palo Alto, CA, USA), NanoDrop (Thermo Fisher Scientific Inc.) and 1% agarose gel electrophoresis before next generation sequencing library preparations. One microgram of total RNA passing the quality test was used for library preparation based on the manufacturer’s protocol of the NEBNext Ultra RNA Library Prep kit for Illumina.

Illumina HiSeq X10 sequencing platform was employed for the library sequencing using a 2×150 bp paired-end (PE) configuration; image analysis and base calling were conducted by the HiSeq Control Software (HCS) + OLB + GAPipeline-1.6 (Illumina) on the HiSeq instrument. The sequences were processed and analyzed by Genewiz (Suzhou, China).

### Differential gene expression analysis

Quality control for the sequencing reads were conducted by removing low quality reads containing adapter, primers and poly-N to generate the clean data by Trimmomatic (v0.30). The reference genome sequences and gene model annotation files of *S. cerevisiae* S288C were downloaded from NCBI https://www.ncbi.nlm.nih.gov/genome/genomes/15 for the reads mapping. Paired-end clean reads were aligned to the reference genome via Hisat2 software (v2.0.1). RPKM (reads per kilo bases per million reads) values were calculated as the indicators of gene expression abundance. DESeq Bioconductor package, a model based on the negative binomial distribution, was used for the differential expression analysis. After adjusted by Benjamini and Hochberg’s approach for controlling the false discovery rate, p-value <0.05 and the absolute value of log2fold change >1were set together to detect differential expressed ones^48^.

### Enrichment analysis

GO-TermFinder was used for identifying Gene Ontology (GO) terms that annotate a list of enriched genes with a significant p-value <0.05. To identify the key metabolic pathways, KOBAS software 2.0 was employed to enrich significant differential expression gene in KEGG pathways.

### RT-qPCR validation

Four candidates of the differentially expressed genes associated with the vitamin B complex biosynthesis pathway were selected to validate the transcriptome data using quantitative real-time PCR (RT-qPCR) and the list of the genes and primers were shown in Table 3. Amplification and detection were undertaken with a Thermal Cycler Dice Real-Time System (Takara Bio., Kyoto, Japan). The reaction mixtures were consisted with 12.5 μL of SYBR Premix Ex Taq II (Takara Bio.), 0.5 μL of each primer (10 μM) and 2 μL of prepared cDNA made up to a total volume of 25 μL. Forty cycles at 95 °C for 10 s and at 55 °C for 10 s and at 72 °C for 10 s after the primary denaturation at 95 °C for 10 s were run for the PCR program. The β-Actin gene *ACT1* was used as an internal gene expression control. The whole experiment was repeated for three times. The value of 2^−ΔΔCT^ was calculated and used as the indicator for the relative expression level of the target genes^49^.

**Table 3:** Primer lists used in RT-qPCR reactions.

Gene name	Gene description	Forward primer (5′-3′)	Reverse primer (5′-3′)	Product (bp)
*SNZ2*	pyridoxal 5′-phosphate synthase	CAAGGATCTAGGTGAGGCTTTG	CTGGATCTCCGCCTTAATCTTG	130
*THI21*	hydroxymethylpyrimidine kinase	ATTTGGCTCGCGGTTATTCC	GCCCGTTGTCCTTAACAGTC	114
*THI6*	thiamine-phosphate diphosphorylase	TATGGCAATTGACGCCGATG	GCCAATGTCTCTACCTCCGA	132
*THI80*	Thiamine pyrophosphokinase	ATCCATCCAAACGAGGATGA	CTATTGGCTGCACCATCTGC	131
*ACT1*	Actin	ACATCGTTATGTCCGGTGGT	CCACCAATCCAGACGGAGTA	142

### Measurements of B-complex vitamins (B_1_, B_6_)

In order to verify the up-regulated genes participating into the metabolic pathway of vitamin B_1_ and B_6_ from the aspect of yeast phenotype, the intracellular contents of the two vitamins were measured by using reverse phase high performance liquid chromatographic (RP-HPLC) method^50^. The prepared yeast cells were re-suspend in 2 mL deionized water and almost complete cell breakage was obtained using a mechanical bead beater (Biospec Products) set at 6000 rpm for four 30 s periods alternating with 30 s intervals on ice bath. Supernatant were collected by centrifugation at 10,000 rpm for 2 min for the chromatographic measurement. About 50 mg each of thiamine hydrochloride (VB_1_) and pyridoxine hydrochloride (VB_6_) standard chemicals was weighed accurately and dissolved in 50 mL 1:1 (v/v) distilled water/ acetonitrile solution thoroughly for the preparation of mixed stock standard solutions. The stock standard solutions were filtered through 0.45 µm membrane filter and diluted with distilled water in the test tubes to yield five series of mixed standard solutions.

The separations and quantitative determination were performed on a Waters 2996 HPLC system (Waters Corp., Milford, MA, USA) consisting of an C18 reverse phase column (Waters X-Terra RP 18 250 × 4.6 mm id., 5 µm particle size), a guard column containing the same material and Waters series 2487 spectrophotometric detector. The wavelength was set at 320 nm to separate all the two metabolites in a single injection. The degassed binary mobile phase consisted of 30% ortho phosphoric acid (Solvent A) and 70% acetonitrile (Solvent B). The flow rate was 1.2 mL/min and the volume injected was 20 µL. Sample peaks were identified by comparing the retention times with those of the standard chemicals. Integration of separated peak area was calculated and the content of each vitamin was determined by using the calibration curve of peak area-concentration relationship. The vitamin contents (μg/ g dry cell weight) in each sample were expressed as the normalized values by dry cell mass weight.

### Measurement of cellular fatty acid composition

Freeze dried yeast cells were weighted and subjected to the lipid extraction according to the literature protocol with slightly modification^51^. Briefly, the cells were disrupted in a MiniBeadbeater-16 (Biospec Products Inc., Bartlesville, OK, USA) according to our previous study^52^. Lipids were extracted from the cell homogenates with the mixed solution of chloroform and methanol (2:1, v/v). Fatty acid methyl esters (FAMEs) were prepared by catalytic reaction of 2% H_2_SO_4_ in anhydrous methanol at 70 °C for 2 h and then the FAMEs solutions were analyzed by gas chromatography (Shimadzu GC-2010 plus), with a QP-5000 mass spectrometer, on an Agilent DB-17 capillary column (30 m length, 0.25 mm inner diameter, 0.25 μm film thickness). FAMEs were identified by comparing the corresponding mass spectra with a spectrum database. The fatty acid composition of each sample was determined based on the integrated peak area for each FAMEs shown as a percentage of the total peak area.

### Spot assay

In order to verify the physiological functions of the increased intracellular contents of vitamin B_1_ and B_6_ as well as the up-regulated expression level of the encoding genes, spot assay of the viable cell counts were conducted. The harvested fresh cells were re-suspended in the MSM medium supplemented with 1.0 g/L PAs, 50 mg/L vitamin B_1_, 50 mg/L vitamin B_6_ and a 1:1(w/w) mixture of the two vitamins, respectively. The negative control without the above supplements was set for the comparison of cell viability. All the samples were cultivated statically at 28 °C for 72 h and then serially diluted. Five microliter of proper dilutions was spotted on the MSM agar plate for viable cell counts.

## Supplementary information

Supplementary information.
