# Supplementary material for: Transcriptome analysis reveals the protection mechanism of proanthocyanidins for Saccharomyces cerevisiae during wine fermentation
Source: Sci Rep. 2020 Apr 21;10:6676. doi: 10.1038/s41598-020-63631-2 (PMC7174367; doi:10.1038/s41598-020-63631-2)
Supplement: Supplementary file 1 — Supplementary information. [file 41598_2020_63631_MOESM1_ESM.pdf]

Supplementary Information

Article title: Transcriptome analysis reveals the protection mechanism of proanthocyanidins for *Saccharomyces cerevisiae* during wine fermentation

Author names: Jingyuan Li[1], Kaili Zhu[1], Hongwei Zhao[2,3,*]

[1] College of Food Science and Engineering, Qingdao Agricultural University, Qingdao, Shandong Province, 266109, China

[2] College of Marine Science and Bioengineering, Qingdao University of Science and Technology, Qingdao, Shandong Province, 266042, China

[3] Shandong Provincial Key Laboratory of Biochemical Engineering, Qingdao, Shandong Province, 266042, China

[*]Corresponding author: Hongwei Zhao

E-mail address of the corresponding author: zhaohw@qust.edu.cn

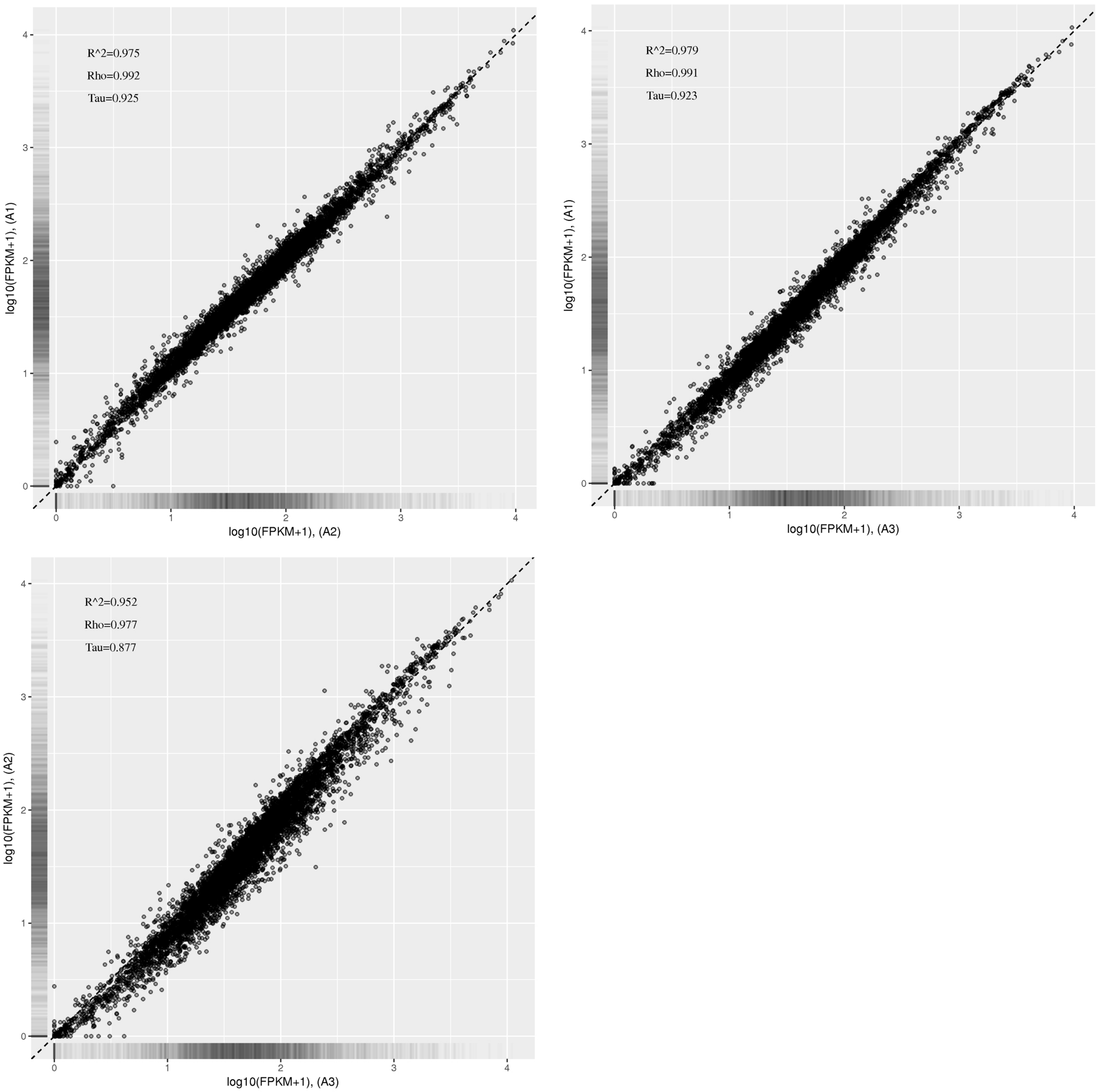

Supplementary Figure S1 Pearson correlation check for the sequencing data in group A. A1, A2, A3 were the three independent biological replicates in group A. The RPKM value for each gene was calculated as per the Materials and methods section. The $\log_{10}$(RPKM+1) were used to calculate the Pearson correlation coefficient R^2. The sequencing data with a coefficient $R^2 > 0.8$ was considered to be high quality.

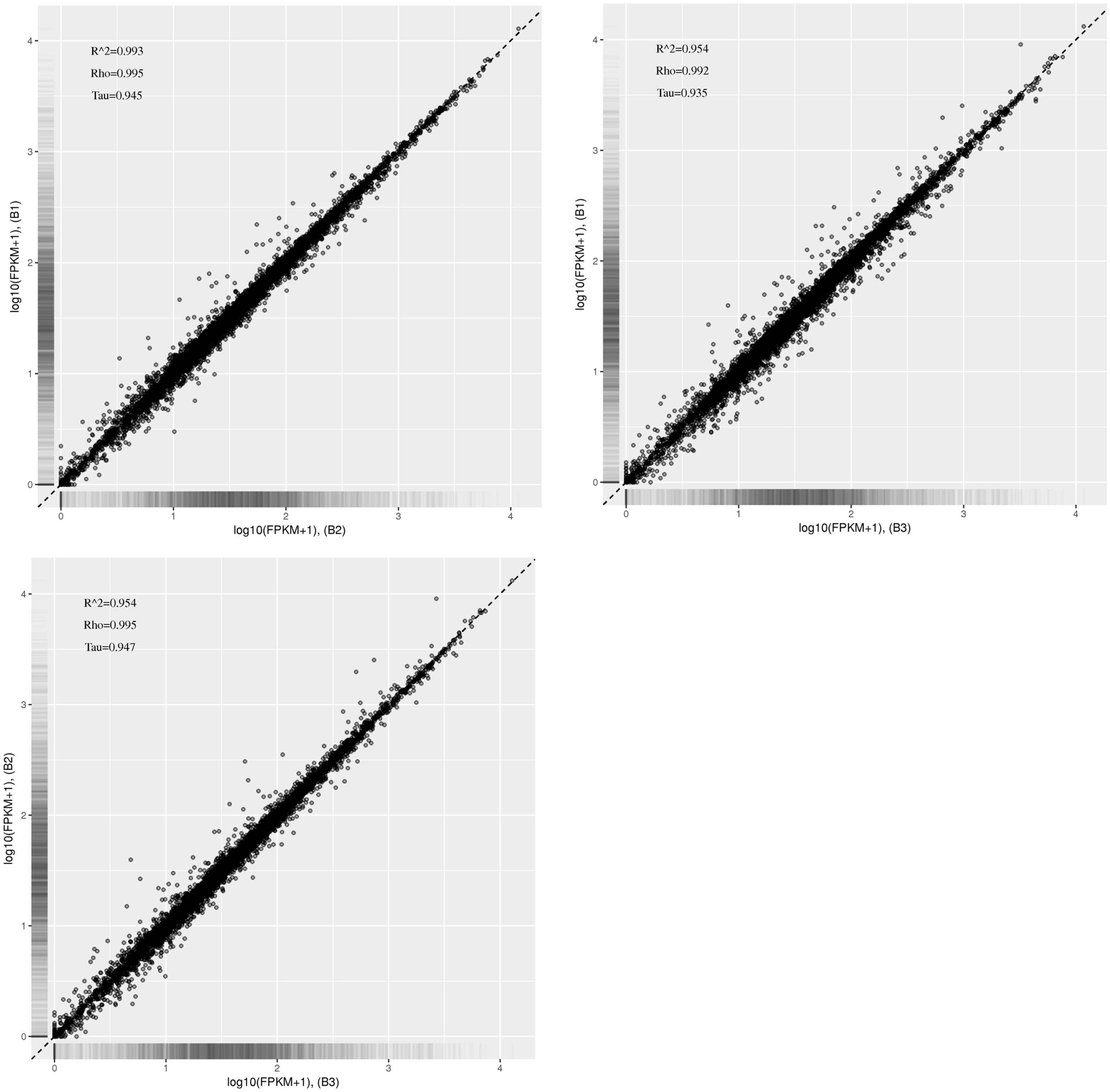

Supplementary Figure S2 Pearson correlation check for the sequencing data in group B. B1, B2, B3 were the three independent biological replicates in group B. The RPKM value for each gene was calculated as per the Materials and methods section. The log_{10}(RPKM+1) were used to calculate the Pearson correlation coefficient $R^{2.}$ The sequencing data with a coefficient $R^2 > 0.8$ was considered to be high quality.

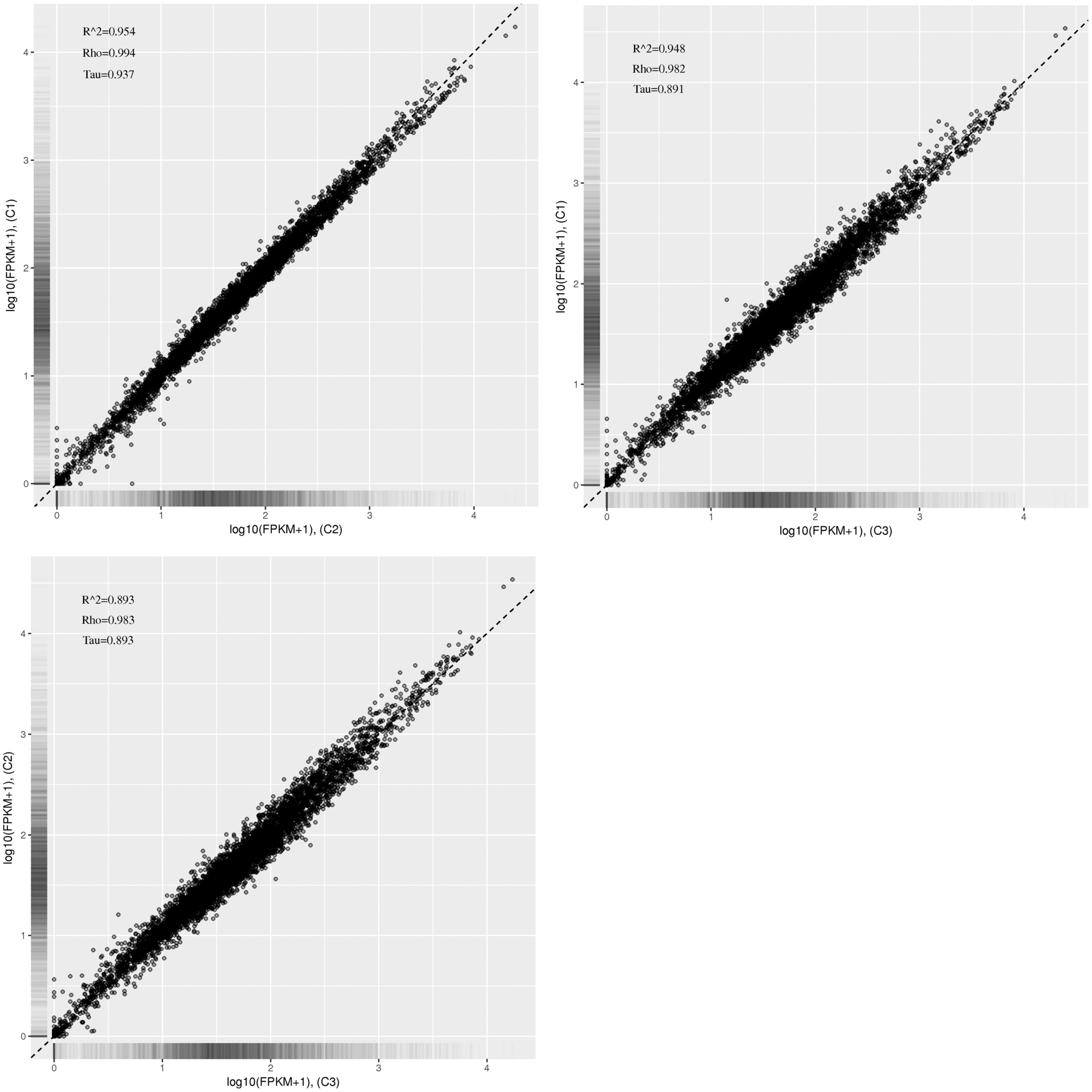

Supplementary Figure S3 Pearson correlation check for the sequencing data in group C. C1, C2, C3 were the three independent biological replicates in group C. The RPKM value for each gene was calculated as per the Materials and methods section. The $\log_{10}$(RPKM+1) were used to calculate the Pearson correlation coefficient R^2. The sequencing data with a coefficient $R^2 > 0.8$ was considered to be high quality.

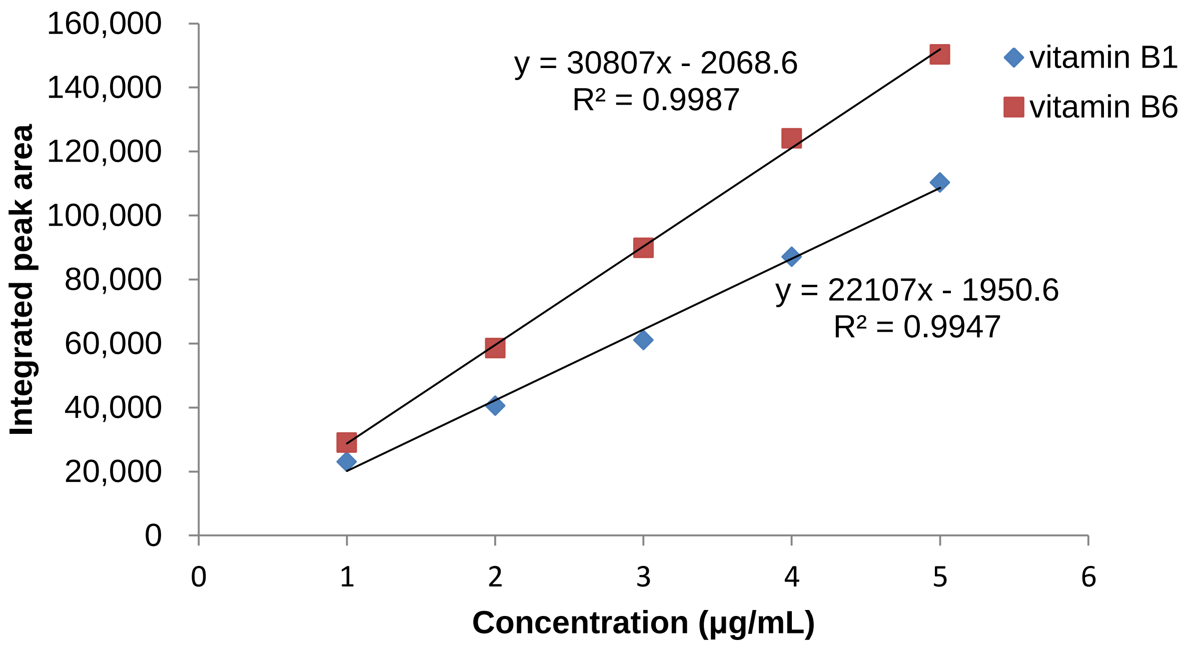

Supplementary Figure S4 The calibration curve of the vitamin B_1 and B_6 standards
